# Small-Scale Woodlot Growers’ Interest in Participating in Bioenergy Market In Rural Ethiopia

**DOI:** 10.1007/s00267-021-01524-4

**Published:** 2021-08-24

**Authors:** Zerihun Nigussie, Atsushi Tsunekawa, Nigussie Haregeweyn, Mitsuru Tsubo, Enyew Adgo, Zemen Ayalew, Steffen Abele

**Affiliations:** 1 Arid Land Research Center, Tottori University, Tottori, Japan; 2 College of Agriculture and Environmental Sciences, Bahir Dar University, Bahir Dar, Ethiopia; 3 International Platform for Dryland Research and Education, Tottori University, Tottori, Japan; 4 Department of Sustainable Regional Management, University of Applied Forest Sciences, Rottenburg, Germany

**Keywords:** Biomass energy, Ethiopia, Drought, *Acacia decurrens*, Biomass supply chain

## Abstract

Production of value-added outputs from biomass residues represents an opportunity to increase the supply of renewable energy in Ethiopia. Particularly, agroforestry could provide biomass residues for improved bioenergy products. The aim of this study was to characterize the interest of growers to provide biomass residues to a hypothetical biomass feedstock market. This study relied on a survey conducted on a sample of 240 farmers. Although the awareness of potential biomass products was generally quite low, a majority of farmers expressed interest in supplying biomass residues, but the level of interest depended on certain individual socio-economic and demographic characteristics. For example, younger and female household heads were found to be more interested in participating in the hypothetical biomass market, as were households with an improved biomass stove, larger land holdings, and higher income levels. In addition, larger households and those that felt less vulnerable to firewood scarcity also expressed more interest. As a whole, the results imply that farmers, particularly those with younger and female heads of households, should be supported with programs tailored to ensure their inclusion in biomass supply chains. Respondents generally preferred farm-gate sales of biomass, so the collecting, baling, and transporting of woody residues need to be properly incentivized or new actors need to be recruited into the supply chain. Providing households with energy-efficient tools such as improved stoves would not only increase demand for biomass products, but also increase the amount of biomass residues that could be supplied to the market instead of used at home.

## Introduction

Globally, the quest for sustainable energy sources has intensified today more than ever before (Dahunsi et al. 2020), because lack of access to clean and modern energy undermines economic development and poverty alleviation efforts (Guta 2020). Rural renewable energy supply is crucial for rural development, worldwide and even more so in sub-Saharan Africa (SSA) (Barbier 2020), where more than 80% of households still burn solid biofuels for cooking and heating (World Bank 2011) and electrification lags significantly far behind other regions of the globe (Dahunsi et al. 2020). In other regions, the supply of electricity and other modern energy sources has been shown to increase rural income and overall well-being (Barbier 2020; Shan et al. 2016), as well as reduce the strain on rural labor systems, in particular for collecting energy sources like firewood or dung (Gwavuya et al. 2012), and allow labor to be allocated more productively (Dinkelman 2011; Narula and Bhattacharyya 2017). Moreover, increasing energy efficiency and reducing emissions from burning fossil fuels or primary biomass energy carriers (wood, charcoal, dung, or agricultural residues) using improved biomass technologies (e.g., stoves) would also improve health conditions, particularly for women and children, by avoiding respiratory diseases from exposure to outdoor and indoor air pollutants (Gwavuya et al. 2012; Hanif 2018; Sulaiman et al. 2020; Tucho and Nonhebel 2015). While some authors (e.g., Hanif 2018; Mulugetta 2008) see the persistent use of biomass resources as energy sources critical and suggest a focus on other renewable energy sources, others (e.g., Berhanu et al. 2017; Tucho and Nonhebel 2015) highlight the high potential of the same, in particular biomass-based energy as affordable energy sources in rural areas, where poverty is an obstacle to purchasing electrical equipment. From the latter’s viewpoint, woody biomass residues may represent an environmentally friendly and cost-effective alternative to fossil fuels for generating affordable biomass-based modern energy products in countries well-endowed with plantation forests (Dahunsi et al. 2020; Nzotcha and Kenfack 2019; Shan et al. 2016; Sulaiman et al. 2020).

Research from Asia has shown that significant cost reductions and energy efficiency gains can be achieved through the use of biomass rather than fossil fuels (Abe et al. 2007; Tareen et al. 2020). In addition, the conversion of previously used raw materials (e.g., residues or dung) and waste into biofuels increases the energy efficiency of the energy carriers themselves even further (Lee et al. 2019; Tucho and Nonhebel 2015). A more recent economic valuation study by Nzotcha and Kenfack (2019) indicated that the use of woody biomass residues for power generation has the potential to enhance the SSA region’s current electricity-generation capacity by 1%. SSA has very low electricity access rates (45.4% in 2018), but it has a high potential for biomass-based energy systems because of the availability of ample raw material resources (IEA 2019). It has, however, experienced major setbacks in the production, distribution, and processing of bio-resources into new bioenergy sources, and the region’s success in this sector depends largely on solving a number of social, economic, technical, and institutional problems (Dahunsi et al. 2020).

There is a longstanding precedent for coupling bioenergy derived from a range of feedstocks (e.g., planted forest and agricultural residues, grass, household wastes, energy crops) and the variety of possible end-uses (e.g., heat, electricity, transport fuel) elsewhere, using conversion techniques like fast pyrolysis, anaerobic digestion, fermentation, gasification, direct combustion, and liquefaction (Bauen et al. 2009; Chan et al. 2019; Lee et al. 2019; Tareen et al. 2020; Tripathi et al. 2019). Woody biomass-derived energy systems are thus considered to have a high development potential in the upcoming decades, both regionally and globally (Lauri et al. 2014; Tareen et al. 2020; Tripathi et al. 2019). As is the case in other SSA countries, Ethiopia has a strong potential to produce biomass-based renewable energy sources (Khatiwada et al. 2019). For example, Ethiopia’s annual exploitable biomass potential is estimated at 141.8 million tons and currently only half of this is exploited (Hailu and Kumsa 2021), and woody biomass constitutes 90% of total energy use, while other energy sources continue to play a minor role (Alemayehu et al. 2020; Berhanu et al. 2017). Consequently, this type of renewable energy resource can be harnessed in the quest to achieving the country’s sustainable development. In this context, new sources of economic value for small-scale producers—as well as social benefits for society—can be unlocked by using unexploited resources of woody biomass residues for producing valuable upgraded bioenergy products. However, in the context of Ethiopia, biomass production as a source of new bioenergy fuels is not yet as common in rural agricultural and agroforestry systems as staple crop production. In addition, there is also a lack of biomass energy plants and production and processing facilities. Consequently, the necessary value chains have yet to be developed and optimized. This implies the need to solve the “chicken and egg problem” of planning and constructing energy plants and processing sites while simultaneously ensuring a sustainable supply of raw materials. Our basic argument here is that there is a complex interaction between (public) institutions like markets, value chains and support schemes on the one hand and individual incentives to participate in the respective markets and value chains on the other. One cannot function without the other, which leads to the aforementioned typical “chicken and egg” state. The study aimed at resolving this problem by determining whether there are individuals who have incentives to participate in the (hypothetical) markets and value chains. At the same time, the study also has the overall goal to determine which institutional incentives may be set to encourage potential participants, by analyzing respective characteristics and constraints of individuals which could be lifted through institutional support.

There seems to be an overall lack of knowledge and research concerning bioenergy value chains, in particular supply chains for biomass in SSA, including Ethiopia, despite the acknowledged large potential of biomass-based energy in the country (Dahunsi et al. 2020; Guta 2012). The present study aimed to characterize the interest that potential small-scale farmers have in supplying woody biomass for a hypothetical biomass market and to create awareness about the possibility of sourcing raw materials for biomass-based bioenergy production from existing small-scale farmers’ *Acacia decurrens* (hereafter “acacia”) tree plantation systems in Ethiopia. The goal was to assess the feasibility of processing woody biomass residues from acacia plantations into value-added products and evaluate establishing a new rural industry and improving the local energy supply, as well as optimizing the utilization of acacia plantations and their products and byproducts. As far as the paper’s contribution to theoretical and methodological aspects is concerned, the goal was also, beyond the above-mentioned feasibility aspect, to provide a ‘blueprint’ on how to research the “chicken and egg” problem. Researches had, on many occasions, have to deal with this kind of problem, as in many economic cases, in particular in developing countries, and while many technical and socio-economic issues have been resolved or at least explained in recent years, problems of the “chicken and egg” type have not yet been resolved.

We hypothesized that woody biomass residues from acacia woodlots may provide commodity benefits, in monetary terms, which exceed the amount a woodlot would offer from the same area in the current system. As a result, small-scale growers could become interested in supplying their harvest residues from acacia plantations, and that, based on this newly available supply, processing plants for emerging modern bioenergy production could be established. We also hypothesized that acacia growers’ interest in supplying woody biomass residues for biomass energy production is based on certain conditions related to individual resource endowments and other social and demographic characteristics. By understanding how acacia growers respond to potential emerging bioenergy markets and identifying the factors that explain their responses to such opportunities, we should be able to anticipate and plan for subsequent individual- and societal-level outcomes. As previous studies indicate, adoption and decision-making processes in the bioenergy sector depend on individual conditions (e.g., attitudes toward risk and other socio-demographic and farm characteristics) (Alemayehu et al. 2020; Wang and Watanabe 2016; Wolde et al. 2016). These conditions and characteristics need to be considered when discussing how small-scale tree plantation systems in Ethiopia can be supported so farmers may join the biomass energy market and supply chain. In addition, small-scale farmers’ interest in participating in a potential new woody biomass residues supply chain depends on the interaction of several factors, as is also the case for other agricultural products. However, the type and magnitude of these variables, to our knowledge, have not been systematically investigated in Ethiopia. Hence, understanding farmers’ ex-ante supply decisions needs due attention by relevant stakeholders (e.g., private businesses, government institutions, private-public partnerships, research institutions, energy sector development programs, international development partners) to optimize the utilization of locally available resources from plantation biomass residue because their implications may extend beyond the farm scale to inform the design, support, and promotion of appropriate interventions relative to farmers’ resources and livelihood settings.

### Contextualizing the Acacia Plantation System

Plantation forestry using fast-growing exotic trees has become a major forestry practice in Ethiopia (Belayneh et al. 2020; Guta 2012; Nigussie et al. 2021). A number of exotic tree species have been promoted in the past several years. Among these, eucalyptus and acacia species appeared the most promising tree species (Belayneh et al. 2020; Nigussie et al. 2017). Particularly in the northwestern highlands of Ethiopia, the farming system seems to be gradually shifting toward the cultivation of acacia and eucalyptus plantations, mainly to produce traditional energy sources like charcoal and firewood (Belayneh et al. 2020; Nigussie et al. 2017; Nigussie et al. 2020).

*A. decurrens*, locally known as ‘*girar*’, is a fast-growing multipurpose tree species native to Australia. It was introduced in the 1990s to central highland areas of Ethiopia to predominantly mitigate urban fuelwood shortages (Nigussie et al. 2017). Around the same time, this species was introduced into the northwestern highlands of Ethiopia. Since the last decade, acacia was well acclimatized in this region. Currently, acacia is the leading exotic tree species grown in woodlot plantations in the northwestern Ethiopia, followed by *Eucalyptus* *camadulensis* (Belayneh et al. 2020; Nigussie et al. 2017; Nigussie et al. 2021; Wondie and Mekuria 2018).

Acacia trees produce goods such as charcoal, fuelwood, construction materials, and animal feed, and provide service functions such as soil fertility replenishment and soil conservation (Nigussie et al. 2017). Traditionally, acacia growers mainly use the stems of the acacia for charcoal production by using a rudimentary earth kiln technology (Nigussie et al. 2021). Acacia trees are usually harvested 4–5 years after planting (Nigussie et al. 2017), when farmers clear-fell woodlots for making charcoal, acacia trees attain a height of 9–15 m. Charcoal is the key traditional bioenergy product derived from acacia stems, and small-scale farmers produce it principally for sale to others. There is virtually no local demand for charcoal as charcoal use is a predominantly urban phenomenon. The charcoal produced by farmers is usually sold to traders, which is then sold to final end users, mainly in major urban areas (e.g., Addis Ababa, Bahir Dar, Gonder, Dessie, Mekelle) (Nigussie et al. 2021). In addition to charcoal from the stems, the woody biomass residues (twigs, branches) represent a significant resource in the acacia-based charcoal supply chain.

In the study watershed, the acacia woody biomass residues (twigs and branches), which are collected after clear-cutting of woodlots, are mainly used as domestic firewood sources for cooking and heating purposes (Nigussie et al. 2021). However, handling of these residues (e.g., collection and transportation) for household domestic use depends on the woodlot’s distance from a residence; the greater the distance, the higher the probability that the residues will be left behind. In addition to its current traditional uses, acacia woody biomass residue has a high energy potential to serve as a feedstock for producing improved bioenergy products (Sette Jr et al. 2020; Tareen et al. 2020) as it has the capability to accumulate large quantities of lignocellulosic biomass within a short span of time (Beckinghausen et al. 2020). The residues could be upgraded into improved bioenergy sources (Tripathi et al. 2019) and further improve the sustainability of the plantation system in terms of generating energy for own use and/or additional income from sales. As this resource is currently less tradable commodity or undervalued in the local market or, sometimes, even left behind the farther away the plantation site (Nigussie et al. 2021), the proposed bioenergy market could draw a considerable supply thereby opening new avenues of income generation and boosting the local economies in a short period. Therefore, with regard to the exploitation of the residual biomass resources for energetic purposes, only twigs and branches could be assumed to be available, whereas, leaves remain on site to maintain site soil nutrients.

## Material and Methods

### Study Site

**Fig. 1:**
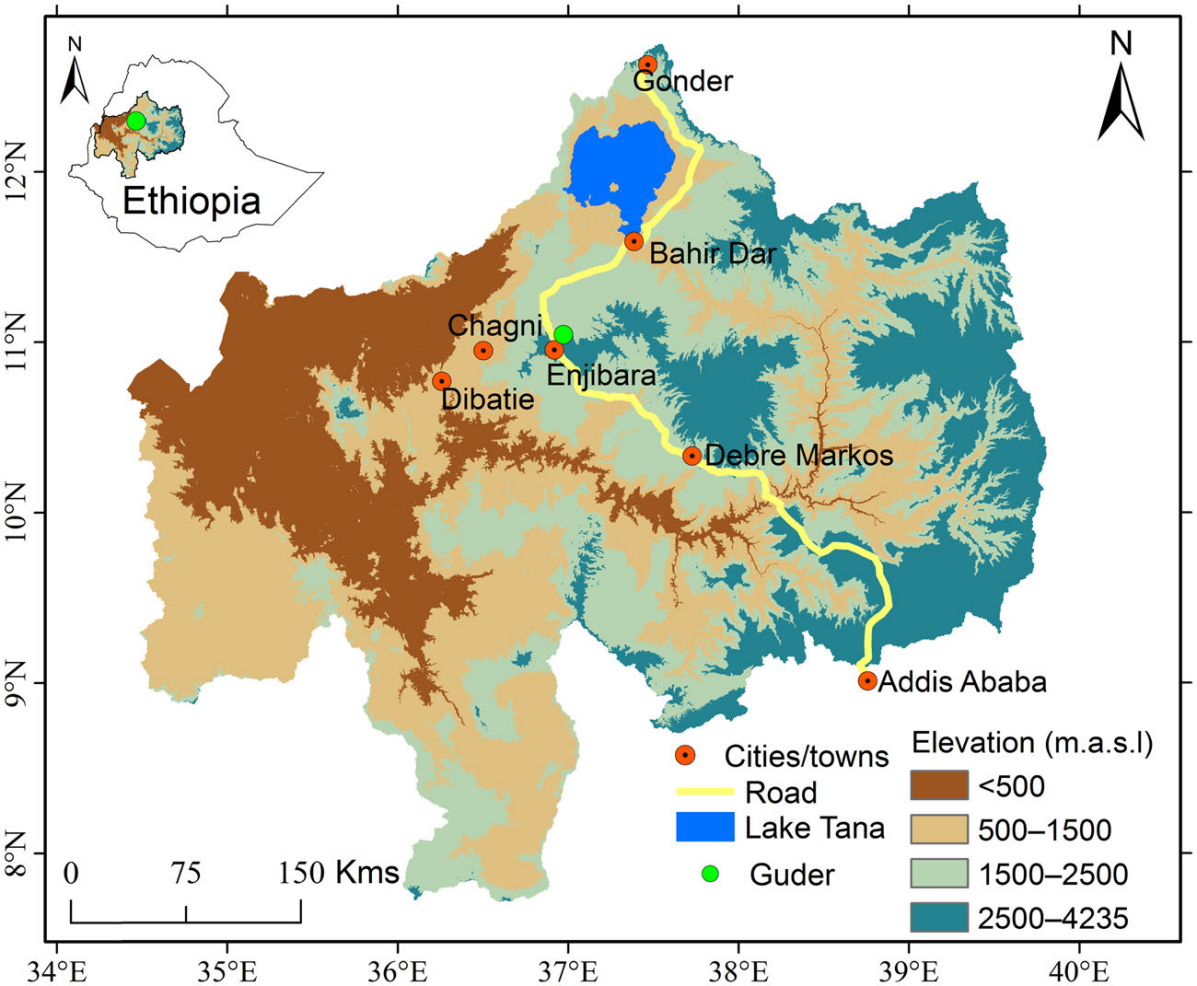
Location map of the study area (USGS 2021)

This study was conducted in the Guder watershed, a hotspot for the exotic fast-growing acacia plantation boom, in the northwestern highlands of the Upper Blue Nile Basin of Ethiopia. Geographically, the Guder watershed is located at 10° 59′ 34′′ 11° 01′ 01′′ N and 36° 54′ 09′′ 36° 55′ 55′′ E (Fig. 1). It is located in Fagita Lekoma District of the Amhara Regional State and covers an area of 741 ha. The elevation ranges from 1800 to 2900 m above sea level, and the area is characterized by a moist subtropical climatic condition. The mean annual minimum and maximum monthly temperatures are 5 °C and 25 °C, respectively. The mean annual total rainfall in the watershed is 2495 mm.

The livelihood of the community is mainly derived from a rain-fed mixed subsistence crop-livestock farming and charcoal making (Abeje et al. 2019; Nigussie et al. 2017; Nigussie et al. 2021). The major staple crops cultivated include teff (*Eragrostis abyssinica*), barley (*Hordeum vulgare*), potato (*Solanum tuberosum*), and wheat (*Triticum vulgare*). The dominant livestock types are cattle, sheep, donkeys, horses, and poultry. The predominant fast-growing exotic species include acacia and *E. camadulensis* (Abebe et al. 2020; Nigussie et al. 2020; Wondie and Mekuria 2018). Clear felling (acacia) and coppice management (*E. camadulensis*) are the common plantation management practices (Abebe et al. 2020). The soil, mainly Acrisols and Leptosols (Abebe et al. 2020), is acidic and severely degraded (Wondie and Mekuria 2018). Acacia has the ability to fix nitrogen contributing to its successful adaptation in acidic soils of the Guder watershed, and very recently it is listed among legume tree species recommended for reclaiming acid soils in Ethiopia (Amede et al. 2019).

### Survey Design, Questionnaire, Sampling, and Data Collection

Survey methods involve data collection using a survey instrument or structured questionnaire. It is a common tool used in social sciences to gain insights into an understanding of household’s decision-making and behavior, in general (Galbraith 2020; Singh et al. 2016; Sovacool et al. 2018; Young et al. 2018); and collecting data that would allow to measure and determine producers’ interest in involving in bioenergy development, in particular (Alemayehu et al. 2020; Convery et al. 2012; Curman et al. 2016; Dulys-Nusbaum et al. 2019; Gowan et al. 2018). This study applied a cross-sectional household survey research design. The investigation was divided into two stages. The first stage was an exploratory study. In January 2020, a series of field appraisals (field observations) and informal interviews with 12 key informants (6 small-scale acacia growers, 3 forest experts, and 3 energy experts) were initially carried out at the study site to gain a better understanding on woody biomass utilization and management practices employed by small-scale acacia growers. Key informants were selected based on their knowledge of the locality. Insights gained from those field observations and informal interviews, coupled with our review of the previous empirical literature, were enabled us to conduct a preliminary assessment of the factors that may determine farmers’ possible participation in a hypothetical biomass feedstock market and formulate the initial survey. In addition, this stage assisted us in putting our quantitative findings in perspective at latter stages.

Following this step, a draft structured survey questionnaire was designed to collate data from acacia growers on a range of topics. The survey questionnaire was organized in three parts. The first part of the questionnaire included questions about household socio-demographic characteristics, resource endowment, and farm and economic profiles. The second part contained questions regarding household’s possession of an improved biomass stove, number of eucalyptus trees, years of acacia farming experience, number of parcels and area occupied by acacia plantations, current biomass uses and plantation sales strategy, and respondent’s knowledge about any type of products that can be produced from acacia’s woody biomass residues.

In the third part of the survey questionnaire, we included a section that informed the participant about the possibility of producing bioenergy products (e.g., briquettes or pellets) from acacia’s woody biomass residues and the markets that could emerge in the near future for these bioenergy feedstocks (i.e., the hypothetical biomass feedstock market scenario). The survey then asked the respondent to indicate: (1) their interest in participating in a bioenergy feedstock market; (2) the relative strength of their interest; (3) their preferred biomass supply method; and (4) their perception about whether local firewood availability would be reduced if a hypothetical feedstock energy market existed. The question on the variable of interest, i.e., the farmer’s interest in participation, was set as a binary choice (yes/no), asking growers if they would consider supplying their woody biomass residues to the proposed feedstock market. In addition, respondents who expressed their interest to participate in the proposed bioenergy market were presented with a question that would indicate the strength of their interest. More specifically, we asked: “How interested would you be in participating in the proposed bioenergy program if it were profitable for you?” on a response scale of 1 (very low) to 5 (very high). The notion of “profitability” was left to be defined by the respondent in order to avoid a potentially confusing pretext. In the absence of any formal bioenergy market for woody biomass in this region, keeping the notion of profitability personal, enabled us to get a response from all respondents, and avoided possible influence of a threshold price that would have been somewhat arbitrarily given by the researchers on their response. The draft questionnaire was then pilot-tested on 20 acacia growers to ensure the clarity and adequacy of the information sought.

The second stage was the formal research. Following the preparation of a final questionnaire, a sampling frame consisting of acacia-growing household heads in villages of the Guder watershed was obtained from the local agriculture office. A systematic random sampling technique was employed to select a total of 240 acacia-growing household heads for the survey. Finally, the questionnaire was administered in face-to-face interviews in February and March 2020. Prior to starting each interview, the respondents were first informed about the research objectives, and then their verbal consent was obtained. The interviews lasted approximately an hour on average.

### Theoretical Approach

The decision-making process of farm households has been investigated in the light of the theoretical framework of the theory of utility. With the theory of utility, what is deemed necessary about utility regarding choice(s) being made is whether a given option has a higher utility than another option. It has been assumed that the farm household’s decisions are the results of rational choices reflecting their utility maximization option. Accordingly, each individual will choose the alternative that gives them the highest utility (McFadden 1973; Mendola 2007).

Assuming they behave in a rational manner, small-scale acacia growers will seek to maximize their utility with respect to the use of their woody biomass residues by selecting their subjective preference from a set of available alternatives, for either supplying their woody biomass residues to the proposed market or using them for something else. To take into account the uncertainties that surround the acacia growers’ decision-making processes, a random utility model was used to determine the factors influencing acacia growers’ interest in participating in the biomass feedstock market. As a result, the utility functions of alternatives can be divided into observed ($U_i$ and $U_j$) and unobserved (${\upvarepsilon}_i$ and ${\upvarepsilon}_j$) parts and additively expressed as:

$$U_i = U\left( {x{^\prime} } \right) + \varepsilon _i\,{{{{{\mathrm{and}}}}}}\,U_j = U\left( {x{^\prime} } \right) + \varepsilon _j$$

where $U_i$ and $U_j$ are the utilities obtained by acacia growers by deciding to participate and not to participate in a biomass supply scheme, respectively; $x^\prime$ is a vector of explanatory variables, and ${\upvarepsilon}_i$ and ${\upvarepsilon}_j$ are the error components of the respective utilities with a normal distribution. The choice of participating $(U_i)$ over not participating $(U_j)$, implies that the utility function is better maximized by deciding to participate than by deciding not to participate.

### Empirical Approach

Discrete choice models are econometric models particularly suited to handle decision-making process that produce discrete or categorical outcomes (Gujarati 2004). When the dependent variable is dichotomous or binary in nature, many studies have traditionally applied the logit or probit models to determine the factors that influence decision-making (Cameroon and Trivedi 2010; Gujarati 2004). The logit and probit models have the advantage of good fitting in case of limited dependent variables. They yield essentially similar results except that probit has a normal cumulative distribution function (i.e., it has a flatter tail) while logit model assumes a logistic distribution of the dependent variable (Cameron and Trivedi 2005; Gujarati 2004). Both the logit and probit models are estimated by maximum likelihood estimation (MLE) (Cameron and Trivedi 2005; Cameroon and Trivedi 2010; Gujarati 2004). There is no compelling reason as such to choose one over the other. As a result, many researchers regard them as nearly interchangeable (Cameron and Trivedi 2005; Gujarati 2004). The choice on which model to use, thus, is largely based on own experience, availability of statistical software’s, and the preference of the researcher. In this study, the binary probit regression model was employed.

The probit model is commonly used to model the relationship between a binary response variable and one or more explanatory variables, which can be either discrete or continuous. The dependent variable takes the value of 1 if an acacia grower indicates an interest to participate in the proposed biomass supply scheme, and 0 otherwise. To empirically implement the model, we assumed that there is a latent (unobserved) variable or unobservable net utility, $y \ast = U_i - U_j$, that generates the observed variable (*y*), which represents a farmer’s utility acquired from participating in the biomass feedstock market and can be specified as described as follows:

$$y = \left\{ {\begin{array}{*{20}{c}} {1,\,{{{{{\mathrm{if}}}}}}\,y^ \ast \, > \; 0\;{{{{{\mathrm{or}}}}}}\,U_i \, > \, U_j} \\ {0,\,{{{{{\mathrm{if}}}}}}\,y^ \ast\, \le 0\;{{{{{\mathrm{or}}}}}}\,U_i \,\le\, U_j} \end{array}} \right.$$

$$\Pr \left( {{{{{{\mathrm{y}}}}}} = 1 \setminus x} \right) = {\Phi}\left( {x{^\prime} \beta } \right),$$

where *y* is the dependent variable, ${\Phi}(.)$ is the standard normal cumulative distribution function, *β* is a *K* × 1 parameter estimates vector, and *x*′ is a 1 × K vector of explanatory variables. A positive sign means that the explanatory variable helps to increase the probability of an acacia grower’s interest to participate in the proposed biomass supply scheme, and a negative sign implies the opposite effect. The probit model can be estimated using the MLE method.

### Data Analyses

Data management was performed in SPSS ver. 23 (IBM Inc., Armonk, NY, USA). Descriptive and econometric techniques were employed to analyze the data collected from the respondents using Stata ver. 15.1 (Stata Corp LP, College Station, TX, USA). Descriptive statistics including percentage, frequency, mean, and standard deviation were used to analyze the data. Aside from descriptive analysis, for the econometric analysis, a binary probit regression model was used to examine the factors influencing acacia growers’ interest in participating in the hypothetical feedstock market. As a robustness check, the linear probability (LPM) and logit model were also estimated to determine whether the probit model results would change substantially (Appendix Table 1).

### Factors Influencing Farmer’s Interest in Bioenergy Development

Farmer’s behavior expressed through their choices in providing woody biomass residues to a hypothetical biomass feedstock market is influenced by a complex set of factors. Based on previous literature, context and locale rationale, the most prevalent factors have been identified and were used as independent variables for further analysis in this study. The explanatory variables included farmer-specific variables (age, gender, household size, level of education, household income) (Curman et al. 2016; Guta 2020; Halder et al. 2014; Hand et al. 2019; Joshi and Mehmood 2011; Qu et al. 2016; Wolde et al. 2016); farm-specific factors (operating farm size, years of acacia farming experience, number of eucalyptus trees owned) (Guta 2020; Hand et al. 2019; Joshi and Mehmood 2011; Mengistu et al. 2016; Qu et al. 2016; Wolde et al. 2016); perception toward risk factor (perceptions about reduced firewood access) (Curman et al. 2016; Halder et al. 2014; Qu et al. 2016); and access to technology factor (possession of improved stove) (Duguma et al. 2014). Therefore, evaluating these factors is important to find out which variables are significant and shape the responsiveness of small-scale acacia growers to the proposed bioenergy program. Definitions of the selected variables included in the probit model, hypotheses of the direction of their influence and their descriptive statistical measures are presented in Table 1.

**Table 1:** Description and summary statistics of the explanatory variables used in the analysis

Variable (unit)	*H*_0_ sign	Mean (SD)
Age of HH (years)		49.47 (11.96)
20–35	+	30.10 (4.26)
36–64	+	49.44 (7.62)
>64	–	69.71 (5.27)
Gender of HH (1 = female, 0 = male)	–	0.20 (0.40)
Household size (no. of members)		5.53 (1.71)
2–5	–	3.90 (1.03)
6–7	+	6.41 (0.49)
>7	+	8.22 (0.64)
Education level of HH (years)	+	3.13 (3.42)
Operating land size (ha)		1.42 (0.69)
<1.10	–	0.76 (0.24)
1.10–1.59	–	1.33 (0.15)
>1.59	+	2.18 (0.56)
Total household cash income ('000 ETB)		39.13 (25.55)
<24	–	17.32 (3.59)
24–42	–	33.27 (5.)
>42	+	66.80 (25.29)
Years of acacia farming experience (years)		8.38 (3.43)
3–8	–	6.36 (1.13)
>8	+	11.36 (3.52)
Household had improved stove (1 = yes, 0 = no)	+	0.33 (0.47)
Number of eucalyptus trees (no. of trees)	+	103.97 (140.89)
Farmer perceived reduced firewood access (1 = yes, 0 = no)	–	0.46 (0.49)

*H*_0_ sign is the predicted direction of the effect

*HH* household head, *SD* standard deviation, *ETB* Ethiopian Birr (At the time of the survey USD 1 ≈ ETB 32)

## Results

### Characteristics of the surveyed respondents

Table 1 summarizes the demographic and socio-economic characteristics of the surveyed respondents. The average age of the respondents in the sample was about 49 years, with most aged between 36 and 64 years (76.25%). The majority of the households (80%) were male-headed. The average household size for sampled households was 5.53 persons, which is a little higher than the national average of 5.34 persons. Most respondents (45.42%) belonged to a household with 6–7 members, whereas ~43% of them belonged to a household with up to five members. The average schooling attained in the sample was a little more than 3 years. The average land-holding size was 1.42 ha (range 0.25–5.13 ha), which is higher than the regional average holdings of 1.15 ha. The average annual income was about ETB 39,131. On average, respondents had a little more than 8 years of acacia farming experience. One-third of the respondents stated possession of improved biomass stoves. The average number of eucalyptus trees owned by the respondents was about 104 trees. Indeed, 46% of the respondents perceived that using acacia residue as a feedstock source for a proposed bioenergy supply program would reduce local firewood availability.

### Awareness about Alternative Biomass Uses

When asked about their familiarity with any type of product that could be produced from acacia biomass residues prior to this survey, the vast majority (232 respondents) indicated that they were unaware of such products (Table 2). The few respondents who reported they were familiar (8) mentioned charcoal and compost as possible byproducts. Only 18% of respondents indicated that they had any experience selling woody biomass residues in the past (Table 2).

**Table 2:** Respondents awareness of new products from biomass residues and their previous experience in selling biomass

Characterstics	Response	*N*	%
Aware of any product that can be produced from residual woody biomass	Yes	8	3.33
	No	232	96.67
Any experience of selling woody biomass residues	Yes	44	18.33
	No	196	81.67

### Interest in participating in the bioenergy program

**Fig. 2:**
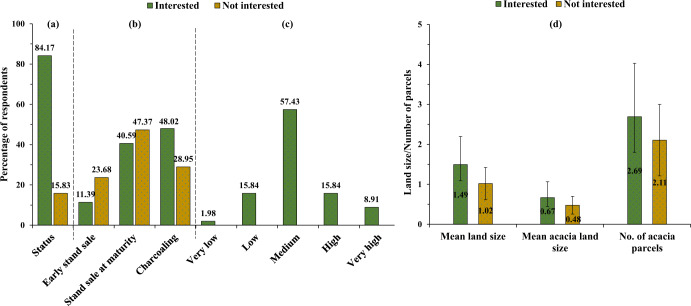
The respondents’: (**a**) interest in participating in the hypothetical supply program, (**b**) current acacia selling strategy, (**c**) level of interest for those who are interested in participating in the hypothetical program, and (**d**) operating land size, acacia plantation size, and no. of acacia parcels

When given a brief informative description about a hypothetical acacia biomass processing program, the majority of the respondents (84%) expressed interest in participating (Fig. 2a). Of these, more than 82% were moderately to highly interested in participating in the proposed market (Fig. 2c).

In the current acacia plantation and biomass use system, the majority of respondents make charcoal (45%) and sell their stands at maturity (41.7%). A similar pattern was observed among those who were interested in participating in the hypothetical program and those who were not (Fig. 2b). Respondents belonging to the interested group possessed, on average, about 46% more acreage than their uninterested counterparts (1.49 vs. 1.02 ha; *p* < 0.001, *t* (238) = –4.0431) (Fig. 2d). The average size of acacia plantation owned by respondents was 0.64 ha, which was fragmented into more than two parcels in 82.5% of cases. Both groups allocated roughly one-half of their acreage for acacia plantation.

When asked about their preffered place for conducting a biomass transaction, most of the respondents who said they would be interested in participating in the hypothetical program (90.59%) stated that the farm gate would be their preferred location. Our informal interviews indicated that the acacia growers would find transporting the biomass themselves to be difficult. If such a market existed, they would rather collect, bale, and store the biomass residues on-farm until the materials could be picked up by the purchaser. This implies that new activities (e.g., biomass aggregation, drying, storage, and transportation) and actors will be needed to supply the residual woody biomass for the proposed feedstock bioenergy production system.

### Model Results

The probit model was estimated to explore the factors that influence small-scale acacia plantation owners’ interest in participating in a hypothetical biomass feedstock supply program. The model results are presented in Table 3. The log-likelihood test for the null hypotheses that all of the coefficients in the model are simultaneously equal to zero indicated that the joint significance of the explanatory variables was highly significant (*p* < 0.0001). In addition, by running the LPM and logit model, qualitatively similar results were found (Appendix Table 1). Thus, our results are robust and consistent.

**Table 3:** Probit model parameter estimates

Variable	Coefficient	Marginal effect
Age of HH (36–64 years, base)		
20–35 years	1.199 (0.570)**	0.207 (0.097)**
>64 years	–0.184 (0.351)	–0.032 (0.061)
HH is female	0.537 (0.292)*	0.093 (0.049)*
Household size (2–5 members, base)		
6–7 members	0.340 (0.300)	0.059 (0.051)
>7 members	0.855 (0.348)**	0.148 (0.058)**
Education level of HH	0.072 (0.049)	0.012 (0.008)
Operating land size (<1.10 ha, base)		
1.10–1.59 ha	0.052 (0.291)	0.009 (0.050)
>1.59 ha	0.872 (0.383)**	0.151 (0.065)**
Total household cash income ('000 ETB) (<24 ETB, base)		
24–42	–0.074 (0.279)	–0.013 (0.048)
>42	0.665 (0.364)*	0.115 (0.062)*
Household had 3–8 years of acacia farming experience	–0.097 (0.268)	–0.017 (0.046)
Household had an improved biomass stove	0.816 (0.347)**	0.141 (0.059)**
Number of eucalyptus trees	0.060 (0.064)	0.010 (0.011)
Farmer did not perceive reduced firewood access	0.529 (0.245)**	0.092 (0.041)**
Constant	–0.888 (0.458)*	
*Log likelihood*	–73.7094	
Likelihood ratio chi-square (14)	62.29	
*Pseudo R*^*2*^	0.297	
*p*	<0.0001	
*Observations*	240	

Numbers in parentheses are standard errors

*HH* household head

*p < 0.10; **p < 0.05

Of the demographic variables, age (20–35 years; *p* < 0.05), household size (>7 members; *p* < 0.05), and gender (female; *p* < 0.10) positively and significantly affected households’ interests in participating in the biomass feedstock supply program. Acreage size (>1.59 ha; *p* < 0.05) and income (>42 ETB; *p* < 0.10) also had a significant positive effect on households’ interest in the program, as did owning an improved biomass stove (*p* < 0.05). Plantation owners who perceived that using biomass residues for other purposes would not reduce firewood availability were more likely to be interested in participating in the biomass supply program (*p* < 0.05).

## Discussion

The relatively high level of interest expressed by small-scale acacia growers to participate in the hypothetical woody biomass residue supply scheme seems to suggest that a latent bioenergy feedstock market “potential” exists in the region. Furthermore, the study showed that respondents had a fairly low knowledge base about any improved bioenergy products that can be produced from acacia woody biomass residues. If a formal market for such resources is to be created, there appears to be a need to address awareness issues among acacia growers through outreach programs not only to enable them to allocate their biomass resources for the best possible uses (i.e., the supply side) but also to make them part of any future demand for such new alternative bioenergy sources (Gowan et al. 2018). In addition, because most respondents were not interested in carrying out activities such as aggregation, baling, drying, and transportation of woody biomass residues, there could also be a need to support either the introduction of additional biomass supply chain actors that could undertake these activities, or the price of biomass has to be set as competitive as possible to incentivize growers to conduct these activities.

Being led by a younger household head (20–35 years old) had a significantly positive effect on the respondent’s interest in participating in a hypothetical biomass feedstock market. This result is consistent with our initial hypothesis as well with results from previous studies (Curman et al. 2016; Hand et al. 2019; Joshi and Mehmood 2011; Proctor and Lucchesi 2012; Qu et al. 2016; Van Dael et al. 2017), who found that younger household heads are more open to trying (and reaping) the benefits of new ventures (e.g., technologies or emerging markets) or have more positive affective and cognitive evaluations of new bioenergy sources. In addition, because this group derives fewer benefits from the current plantation system than older farmers (Nigussie et al. 2020; Nigussie et al. 2021), the results may also imply that younger farmers have higher expectations of spillover benefits (e.g., employment opportunities) from any additional demand for services in the biomass supply chain. A plausible explanation that can be advanced for this view is that younger farmers, who are healthier, have a higher ability, and thus they are more likely to foresee and show interest, to partake in any off-farm wage employment options of the proposed bioenergy program than older farmers (Proctor and Lucchesi 2012).

Small-scale farmers’ ability to participate in tree planting depends on the size of land a household owns (Amare et al. 2019; Nigussie et al. 2017). Operating acreage size was used here as a proxy for the household’s ability to allocate more land to acacia plantations. Households that had a relatively larger land holding were more likely to allot more land to the cultivation of acacia (Nigussie et al. 2017), which may enable them to produce enough firewood to satisfy their domestic needs as well as sell surplus biomass in a hypothetical market. Holding all else constant, this would probably mean that these households have a greater incentive to participate in a biomass feedstock market as compared to those who are poorly endowed, simply because they have more resources to take advantage of economies of size in terms of producing biomass and selling it into the market (Curman et al. 2016; Hand et al. 2019; Qu et al. 2016; Stjepan et al. 2015). Conversely, according to key informants, the availability of such a market may also serve as an incentive for smaller land owners (e.g., land-poor farmers and women) to engage in collecting woody biomass from nearby open access forests (e.g., communal and natural forests), as has been the case for generations, instead of relying only on biomass from privately owned woodlots.

The share of female acacia growers in our sample was small, but the findings indicated that female-headed households were more likely to have an interest in participating in a biomass feedstock market as compared to their male counterparts. According to key informants, this finding could be partially related to the fact that, in the current acacia plantation system, after a woodlot is harvested, charcoal is considered to be part of a male’s domain, whereas women typically enjoy the use and access to woody biomass residues for firewood or other purposes. This could give women the authority to manage, use, or dispose of these residues, which may in turn potentially stimulate them to consider supplying the woody residues to a biomass feedstock market. Another possible explanation is that they may consider themselves as benefiting the most from access to any additional bioenergy products produced from such a scheme.

As expected, economic characteristics, such as income, had a significant positive effect on impacting growers’ interest in participating in a hypothetical feedstock supply market. This finding suggests that acacia growers belonging to a higher-earning category, holding all else constant, generally were more likely to favor such biomass supply initiatives. It is very likely that wealthier farmers, either through their larger land holdings or their greater capacity to buy acacia stands from resource-poor farmers, could commit more acreage to cultivation of acacia plantations (Nigussie et al. 2017; Nigussie et al. 2020). This, in turn, would allow them to produce enough woody biomass residues for self-consumption as well as having the resources to collect and prepare biomass for selling to the hypothetical biomass program.

Firewood is the main source of primary energy in rural Ethiopia (Berhanu et al. 2017; Duguma et al. 2014; Guta 2012; Villamor et al. 2020), and the lack of improved technologies represents one of the challenges in efficient biomass resource use (Duguma et al. 2014). An increase in the share of rural households that have more efficient biomass stoves would contribute to increased energy efficiency, and consequently more biomass is available for other purposes (Gebreegziabher et al. 2017; Villamor et al. 2020). Consistent with a priori expectations, the positive coefficient on possession of an improved biomass stove indicated that ownership of this type of energy-efficient technology has positive implications on acacia growers’ interest in participating in the hypothetical biomass feedstock market. Using improved biomass stove technologies allows rural households to reduce the quantity of self-consumed woody biomass residues by 20–56% (Duguma et al. 2014). This may in turn stimulate their interest in supplying biomass residues that would otherwise be used for self-consumption or left behind to the feedstock program. Moreover, owning and using improved biomass energy-based equipment was found to raise the awareness for opportunities in this market segment in the United Kingdom (Convery et al. 2012). Perceived risk is also an important aspect of any potential emerging biomass supply market, and as expected, the acacia growers who perceived they were less vulnerable to a potential firewood shortage were more likely to express interest in participating in the program. However, this perception of firewood availability could be related to a perception of improved energy efficiency resulting from improved stove ownership and warrants further study.

## Conclusion

Our findings not only have implications for policy but also for measures and actions taken by private actors along bioenergy value chains to support the primary supply of acacia woody biomass residues. Younger and wealthier farmers are more likely to be interested in participating in a hypothetical biomass feedstock market, which implies that these socio-economic groups have the resources (e.g., labor, land) to engage in collecting the biomass—which is a kind of “add-on” activity, creating additional income. This implies that the less endowed farmers could also be integrated into the local supply chain by supporting them in gathering, drying, and baling. The same holds for female-led households, for whom those bioenergy-related value chain activities could be a welcome source of additional income, but they should be supported in case they face resource constraints. Support could include the provision of information and financing of biomass collection activities (such as wage support or credits to pay labor), but also assistance with logistics, given the fact that respondents preferred to have their biomass bought and sold at the farm gate. Policies that strengthen intermediaries along the chain (e.g., traders) would also support the aggregation and transportation of biomass to the market, as well as maintain linkages along the chain (Helliwell et al. 2020).

Biomass feedstock logistics systems to support the primary producers could also provide employment and income opportunities in rural areas if supported by appropriate policies and financial institutions, particularly to those who currently derive less benefit from the plantation system, such as women and younger and poorer farmers (Nigussie et al. 2020). The facts, that households owning an improved stove tended to be more interested in supplying biomass to the feedstock value chain, as well as households who seem to have good access to firewood and thus energy, indicate that the more efficient the rural energy system is, the more biomass would be available to process instead of being burned in less efficient, but cheaper stove or fireplace systems. This implies that policies should support efficient household energy systems in rural areas, which would create a positive, virtuous circle of increasing and growing rural energy supply.

The potential supply of biomass being dependent on resource endowment, like land and labor, and the fact that the respective farm and household characteristics are quite diverse, it may also be critical to look at community or collective schemes to enable the less favored groups to participate in the value chain.

## Supplementary information

Appendix Table 1

## References

[CR1] Abe H, Katayama A, Sah BP, Toriu T, Samy S, Pheach P, Adams MA, Grierson PF (2007). Potential for rural electrification based on biomass gasification in Cambodia. Biomass Bioenergy.

[CR2] Abebe G, Tsunekawa A, Haregeweyn N, Takeshi T, Wondie M, Adgo E, Masunaga T, Tsubo M, Ebabu K, Berihun ML (2020). Effects of land use and topographic position on soil organic carbon and total nitrogen stocks in different agro-ecosystems of the Upper Blue Nile Basin. Sustainability.

[CR3] Abeje MT, Tsunekawa A, Haregeweyn N, Nigussie Z, Adgo E, Ayalew Z, Tsubo M, Elias A, Berihun D, Quandt A (2019). Communities’ livelihood vulnerability to climate variability in Ethiopia. Sustainability.

[CR4] Alemayehu AG, Gebreeyesus A, Palladino G, Setti M (2020). Behavioral precursors in the innovation-decision process: the case of bioenergy in Ethiopia. Energy Strategy Rev.

[CR5] Amare D, Wondie M, Mekuria W, Darr D (2019). Agroforestry of smallholder farmers in Ethiopia: practices and benefits. Small-scale Forestry.

[CR6] Amede T, Schulz S, Warner J, Tefera S (2019). Managing acid soils for reclaiming livelihoods in Ethiopia.

[CR7] Barbier EB (2020). Is green rural transformation possible in developing countries?. World Dev.

[CR8] Bauen A, Berndes G, Junginger M, Londo M, Vuille F, Ball R, Bole T, Chudziak C, Faaij A, Mozaffarian H (2009). Bioenergy: a sustainable and reliable energy source. A review of status and prospects. IEA Bioenergy: ExCo.

[CR9] Beckinghausen A, Reynders J, Merckel R, Wu YW, Marais H, Schwede S (2020). Post-pyrolysis treatments of biochars from sewage sludge and A. mearnsii for ammonia (NH4-n) recovery. Appl Energy.

[CR10] Belayneh Y, Ru G, Guadie A, Teffera ZL, Tsega M (2020). Forest cover change and its driving forces in Fagita Lekoma District, Ethiopia. J Forestry Res.

[CR11] Berhanu M, Jabasingh SA, Kifile Z (2017). Expanding sustenance in Ethiopia based on renewable energy resources – A comprehensive review. Renew Sustain Energy Rev.

[CR12] Cameron AC, Trivedi PK (2005). Microeconometrics: methods and applications.

[CR13] Cameroon A, Trivedi P (2010). Microeconometrics using stata–revised edition.

[CR14] Chan YH, Cheah KW, How BS, Loy ACM, Shahbaz M, Singh HKG, Shuhaili AFA, Yusup S, Ghani WAWAK, Rambli J (2019). An overview of biomass thermochemical conversion technologies in Malaysia. Sci Total Environ.

[CR15] Convery I, Robson D, Ottitsch A, Long M (2012). The willingness of farmers to engage with bioenergy and woody biomass production: A regional case study from Cumbria. Energy Policy.

[CR16] Curman M, Posavec S, Malovrh ŠP (2016). Willingness of private forest owners to supply woody biomass in Croatia. Small Scale For.

[CR17] Dahunsi S, Fagbiele O, Yusuf E (2020). Bioenergy technologies adoption in Africa: a review of past and current status. J Clean Prod.

[CR18] Dinkelman T (2011). The effects of rural electrification on employment: new evidence from South Africa. Am Economic Rev.

[CR19] Duguma LA, Minang PA, Freeman OE, Hager H (2014). System wide impacts of fuel usage patterns in the Ethiopian highlands: potentials for breaking the negative reinforcing feedback cycles. Energy Sustain Dev.

[CR20] Dulys-Nusbaum E, Klammer SS, Swinton SM (2019). How willing are different types of landowner to supply hardwood timber residues for bioenergy?. Biomass Bioenergy.

[CR21] Galbraith E (2020). Earth System Economics: a bio-physical approach to the human component of the Earth System. Earth Syst Dynam Discuss.

[CR22] Gebreegziabher Z, Van Kooten GC, Van Soest DP (2017). Technological innovation and dispersion: Environmental benefits and the adoption of improved biomass cookstoves in Tigrai, northern Ethiopia. Energy Econ.

[CR23] Gowan CH, Kar SP, Townsend PA (2018). Landowners’ perceptions of and interest in bioenergy crops: Exploring challenges and opportunities for growing poplar for bioenergy. Biomass Bioenergy.

[CR24] Gujarati DN (2004) Basic econometrics. 4th ed. The Mc-Graw Hill

[CR25] Guta D (2012). Assessment of biomass fuel resource potential and utilization in Ethiopia: sourcing strategies for renewable energies. Int J Renew Energy Res.

[CR26] Guta DD (2020). Determinants of household use of energy-efficient and renewable energy technologies in rural Ethiopia. Technol Soc.

[CR27] Gwavuya S, Abele S, Barfuss I, Zeller M, Müller J (2012). Household energy economics in rural Ethiopia: a cost-benefit analysis of biogas energy. Renew Energy.

[CR28] Hailu AD, Kumsa DK (2021). Ethiopia renewable energy potentials and current state. AIMS Energy.

[CR29] Halder P, Paladinić E, Stevanov M, Orlović S, Hokkanen TJ, Pelkonen P (2014). Energy wood production from private forests–nonindustrial private forest owners׳ perceptions and attitudes in Croatia and Serbia. Renew Sustain Energy Rev.

[CR30] Hand AM, Bowman T, Tyndall JC (2019). Influences on farmer and rancher interest in supplying woody biomass for energy in the US Northern Great Plains. Agrofor Syst.

[CR31] Hanif I (2018). Energy consumption habits and human health nexus in Sub-Saharan Africa. Environ Sci Pollut Res.

[CR32] Helliwell R, Seymour S, Wilson P (2020). Neglected intermediaries in bioenergy straw supply chains: Understanding the roles of merchants, contractors and agronomists in England. Energy Res Soc Sci.

[CR33] IEA (2019) SDG7: data and projections: access to affordable, reliable, sustainable and modern energy for all. Flagship report—November 2019. https://www.iea.org/reports/sdg7-data-and-projections/access-to-electricity

[CR34] Joshi O, Mehmood SR (2011). Factors affecting nonindustrial private forest landowners’ willingness to supply woody biomass for bioenergy. Biomass Bioenergy.

[CR35] Khatiwada D, Purohit P, Ackom EK (2019). Mapping bioenergy supply and demand in selected least developed countries (LDCs): Exploratory assessment of modern bioenergy’s contribution to SDG7. Sustainability.

[CR36] Lauri P, Havlík P, Kindermann G, Forsell N, Böttcher H, Obersteiner M (2014). Woody biomass energy potential in 2050. Energy Policy.

[CR37] Lee SY, Sankaran R, Chew KW, Tan CH, Krishnamoorthy R, Chu D-T, Show P-L (2019). Waste to bioenergy: a review on the recent conversion technologies. BMC Energy.

[CR38] McFadden D, Zarembka P (1973). Conditional logit analysis of qualitative choice behavior. Front Econ.

[CR39] Mendola M (2007). Farm household production theories: a review of “Institutional” and “Behavioral” responses. Asian Dev Rev.

[CR40] Mengistu MG, Simane B, Eshete G, Workneh TS (2016). The environmental benefits of domestic biogas technology in rural Ethiopia. Biomass Bioenergy.

[CR41] Mulugetta Y (2008). Human capacity and institutional development towards a sustainable energy future in Ethiopia. Renew Sustain Energy Rev.

[CR42] Narula SA, Bhattacharyya S (2017). Off-grid electricity interventions for cleaner livelihoods: a case study of value chain development in Dhenkanal district of Odisha. J Clean Prod.

[CR43] Nigussie Z, Tsunekawa A, Haregeweyn N, Adgo E, Nohmi M, Tsubo M, Aklog D, Meshesha DT, Abele S (2017). Factors affecting small-scale farmers’ land allocation and tree density decisions in an *Acacia decurrens*-based *taungya* system in Fagita Lekoma District, North-Western Ethiopia. Small Scale For.

[CR44] Nigussie Z, Tsunekawa A, Haregeweyn N, Adgo E, Tsubo M, Ayalew Z, Abele S (2020). Economic and financial sustainability of an *Acacia decurrens*-based *taungya* system for farmers in the Upper Blue Nile Basin, Ethiopia. Land Use Policy.

[CR45] Nigussie Z, Tsunekawa A, Haregeweyn N, Tsubo M, Adgo E, Ayalew Z, Abele S (2021). The impacts of *Acacia decurrens* plantations on livelihoods in rural Ethiopia. Land Use Policy.

[CR46] Nzotcha U, Kenfack J (2019). Contribution of the wood-processing industry for sustainable power generation: Viability of biomass-fuelled cogeneration in Sub-Saharan Africa. Biomass Bioenergy.

[CR47] Proctor F, Lucchesi V (2012). Small-scale farming and youth in an era of rapid rural change.

[CR48] Qu M, Lin Y, Liu C, Yao S, Cao Y (2016). Farmers׳ perceptions of developing forest based bioenergy in China. Renew Sustain Energy Rev.

[CR49] Sette CR, de Moraes MDA, Coneglian A, Ribeiro RM, Hansted ALS, Yamaji FM (2020). Forest harvest byproducts: Use of waste as energy. Waste Manag.

[CR50] Shan M, Li D, Jiang Y, Yang X (2016). Re-thinking china’s densified biomass fuel policies: Large or small scale?. Energy Policy.

[CR51] Singh C, Dorward P, Osbahr H (2016). Developing a holistic approach to the analysis of farmer decision-making: Implications for adaptation policy and practice in developing countries. Land Use Policy.

[CR52] Sovacool BK, Axsen J, Sorrell S (2018). Promoting novelty, rigor, and style in energy social science: towards codes of practice for appropriate methods and research design. Energy Res Soc Sci.

[CR53] Stjepan P, Mersudin A, Dženan B, Nenad P, Makedonka S, Dane M, Špela PM (2015). Private forest owners’ willingness to supply woody biomass in selected South-Eastern European countries. Biomass Bioenergy.

[CR54] Sulaiman C, Abdul-Rahim A, Ofozor CA (2020). Does wood biomass energy use reduce CO_2_ emissions in European Union member countries? Evidence from 27 members. J Clean Prod.

[CR55] Tareen WUK, Dilbar MT, Farhan M, Ali Nawaz M, Durrani AW, Memon KA, Mekhilef S, Seyedmahmoudian M, Horan B, Amir M (2020). Present status and potential of biomass energy in Pakistan based on existing and future renewable resources. Sustainability.

[CR56] Tripathi N, Hills CD, Singh RS, Atkinson CJ (2019). Biomass waste utilisation in low-carbon products: harnessing a major potential resource. npj Clim Atmos Sci.

[CR57] Tucho GT, Nonhebel S (2015). Bio-wastes as an alternative household cooking energy source in Ethiopia. Energies.

[CR58] USGS (2021) DEM from U.S. geological survey earth explorer database. https://earthexplorer.usgs.gov/ Accessed 20 July 2021

[CR59] Van Dael M, Lizin S, Swinnen G, Van Passel S (2017). Young people’s acceptance of bioenergy and the influence of attitude strength on information provision. Renew Energy.

[CR60] Villamor G, Guta D, Mirzabaev A (2020). Gender specific differences of smallholder farm households perspective of food-energy-land nexus frameworks in Ethiopia. Front Sustain Food Syst.

[CR61] Wang L, Watanabe T (2016). Factors affecting farmers’ risk perceptions regarding biomass supply: A case study of the national bioenergy industry in northeast China. J Clean Prod.

[CR62] Wolde B, Lal P, Alavalapati J, Burli P, Munsell J (2016). Factors affecting forestland owners’ allocation of non-forested land to pine plantation for bioenergy in Virginia. Biomass Bioenergy.

[CR63] Wondie M, Mekuria W (2018). Planting of Acacia decurrens and dynamics of land cover change in Fagita Lekoma District in the Northwestern Highlands of Ethiopia. Mt Res Dev.

[CR64] World Bank (2011) Wood-based biomass energy development for Sub-Saharan Africa: Issues and approaches. Washington, DC

[CR65] Young JC, Rose DC, Mumby HS, Benitez-Capistros F, Derrick CJ, Finch T, Garcia C, Home C, Marwaha E, Morgans C (2018). A methodological guide to using and reporting on interviews in conservation science research. Methods Ecol Evolution.

