# Supplementary material for: Small-Scale Woodlot Growers’ Interest in Participating in Bioenergy Market In Rural Ethiopia
Source: Environ Manage. 2021 Aug 24;68(4):553–65. doi: 10.1007/s00267-021-01524-4 (PMC8417006; doi:10.1007/s00267-021-01524-4)
Supplement: Supplementary file 1 — Appendix Table 1 [file 267_2021_1524_MOESM1_ESM.docx]

**Appendix**

Appendix Table 1. LPM and logit model parameter estimates of the factors influencing acacia grower’s interest in participating in a hypothetical biomass energy market.

| **Variable** | **LPM** |  | **Logit** | |
| --- | --- | --- | --- | --- |
|  | **Coefficient** |  | **Coefficient** | **Marginal Effect** |
| Age of HH (36–64 years, base) |  |  |  |  |
| 20–35 years | 0.166  (0.058)*** |  | 2.269  (1.146)** | 0.216  (0.106)** |
| >64 years | -0.033  (0.082) |  | -0.289  (0.630) | -0.028  (0.060) |
| HH is female | 0.156  (0.074)** |  | 0.894  (0.510)* | 0.085  (0.047)* |
| Household size (2–5 members, base) |  |  |  |  |
| 6–7 members | 0.038  (0.057) |  | 0.558  (0.539) | 0.053  (0.051) |
| >7 members | 0.118  (0.057)** |  | 1.565  (0.639)** | 0.149  (0.058)*** |
| Education level of HH | 0.008  (0.005) |  | 0.128  (0.089) | 0.012  (0.008) |
| Operating land size (<1.10 ha, base) |  |  |  |  |
| 1.10–1.59 ha | 0.030  (0.062) |  | 0.178  (0.503) | 0.017  (0.048) |
| >1.59 ha | 0.113  (0.060)* |  | 1.711  (0.757)** | 0.163  (0.070)** |
| Total household cash income ('000 ETB) (<24 ETB, base) |  |  |  |  |
| 24–42 | 0.024  (0.063) |  | -0.133  (0.492) | -0.013  (0.047) |
| >42 | 0.104  (0.053)** |  | 1.355  (0.723)* | 0.129  (0.067)* |
| Household had 3–8 years of acacia farming experience | 0.007  (0.046) |  | -0.139  (0.495) | -0.013  (0.047) |
| Household had an improved biomass stove | 0.103  (0.043)** |  | 1.640  (0.700)** | 0.156  (0.065)** |
| Number of eucalyptus trees | 0.008  (0.013) |  | 0.099  (0.115) | 0.009  (0.011) |
| Farmer did not perceive reduced firewood access | 0.078  (0.044)* |  | 0.955  (0.442)** | 0.091  (0.041)** |
| Constant | 0.422  (0.098)*** |  | -1.649  (0.814)** |  |
| *R^2^* | 0.21 |  |  |  |
| *Log likelihood* |  |  | -73.0813 |  |
| Likelihood ratio chi-square (14) |  |  | 63.55 |  |
| *Pseudo R^2^* |  |  | 0.303 |  |
| *p* |  |  | <0.0001 |  |
| *Observations* | 240 |  | 240 |  |

Note: Numbers in parentheses are robust standard errors for LPM and standard errors for logit model; * *p* < 0.10; ** *p* < 0.05; *** *p* < 0.01.
